# Supplementary material for: Aragonite lithium/magnesium as an indicator of calcification media saturation state in marine calcifiers
Source: Commun Earth Environ. 2025 Nov 28;6(1):984. doi: 10.1038/s43247-025-02945-3 (PMC12662775; doi:10.1038/s43247-025-02945-3)
Supplement: Supplementary file 2 — Supplementary Figs. and tables [file 43247_2025_2945_MOESM2_ESM.pdf]

Aragonite Li/Mg as an indicator of calcification media saturation state in marine calcifiers

Cristina Castillo Alvarez[1,2], Edmund Hathorne[3], Matthieu Clog[4], Adrian Finch[1], Roland Kröger[5], Kirsty Penkman[6], Nicola Allison[1,2*]

[1] School of Earth and Environmental Sciences, University of St Andrews, St Andrews, KY16 9TS, UK

[2] Scottish Ocean Institute, University of St Andrews, St Andrews KY 16 8LB, UK

[3] GEOMAR, Helmholtz Centre for Ocean Research Kiel, Germany

[4] SUERC, University of Glasgow, UK

[5] School of Physics, Engineering and Technology, University of York, York, UK

[6] Department of Chemistry, University of York, York, UK

*Corresponding author, email: na9@st-andrews.ac.uk, ORCID: 0000-0003-3720-1917

Supplementary data

Supplementary Figure 1. Relationship between solution pH and Ω_{Ar} in experiments conducted over variable temperature.

Supplementary Figure 2. $D_{Li/Ca}$ as a function of $[HCO_3^-]$ in precipitations conducted at 25°C.

Supplementary Figure 3. Comparison of precipitation rate versus a) $D_{Mg/Ca}$, b) $D_{LI/Ca}$ and c) $D_{Li/Mg}$ relationships in this and previous studies[1,2]. $D_{Li/Mg}$ in c) are calculated from the aragonite Li/Mg reported by Brazier et al[2], combined with their estimates of water [Li] and [Mg].

Supplementary Table 1. Details of precipitations, seawater and solid chemistry and distribution coefficients in experiments at 25°C with and without amino acid (additive).

Supplementary Table 2. Details of precipitations, seawater and solid chemistry and distribution coefficients in experiments at variable temperature. nd = not determined.

Supplementary Table 3. Expected and measured seawater metal concentrations in replicate IAPSO analyses in each analytical run.

Supplementary Table 4. Values of $CaCO_3$ reference materials analysed with aragonite samples in each run.

Supplementary Table 5. Equations describing $D_{Me/Me}$ as a function of aragonite growth rate (R, µmol m^{-2} h^{-1}) and pH (total scale) in precipitations conducted at 25°C in the presence of 2 mM aspartic acid, glutamic acid or glycine. Coefficients of determination (r^2) and p values for each equation coefficient are shown. p values ≥0.05 are highlighted in bold. Standard errors of equation coefficients and intercepts are included in brackets.

Supplementary Figure 1. Relationship between solution pH and Ω_{Ar} in experiments conducted over variable temperature.

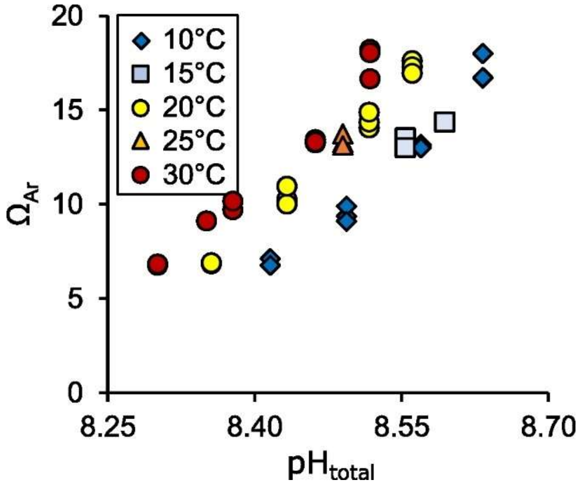

Supplementary Figure 2. $D_{Li/Ca}$ as a function of $[HCO_3^-]$ in precipitations conducted at 25°C.

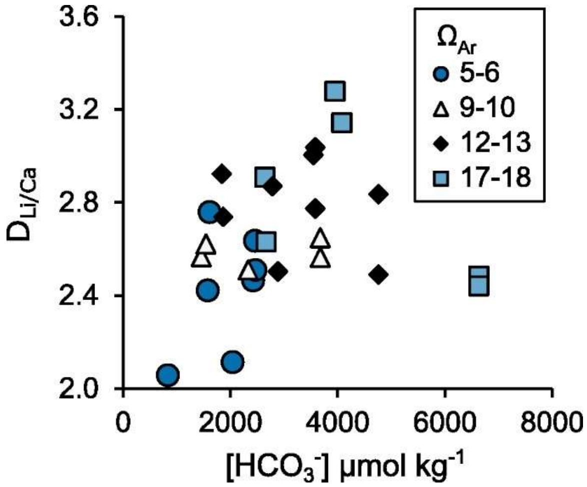

Supplementary Figure 3. Comparison of precipitation rate versus a) $D_{Mg/Ca}$, b) $D_{Li/Ca}$ and c) $D_{Li/Mg}$ relationships in this and previous studies[1,2]. $D_{Li/Mg}$ in c) are calculated from the aragonite Li/Mg reported by Brazier et al[2], combined with their estimates of water [Li] and [Mg].

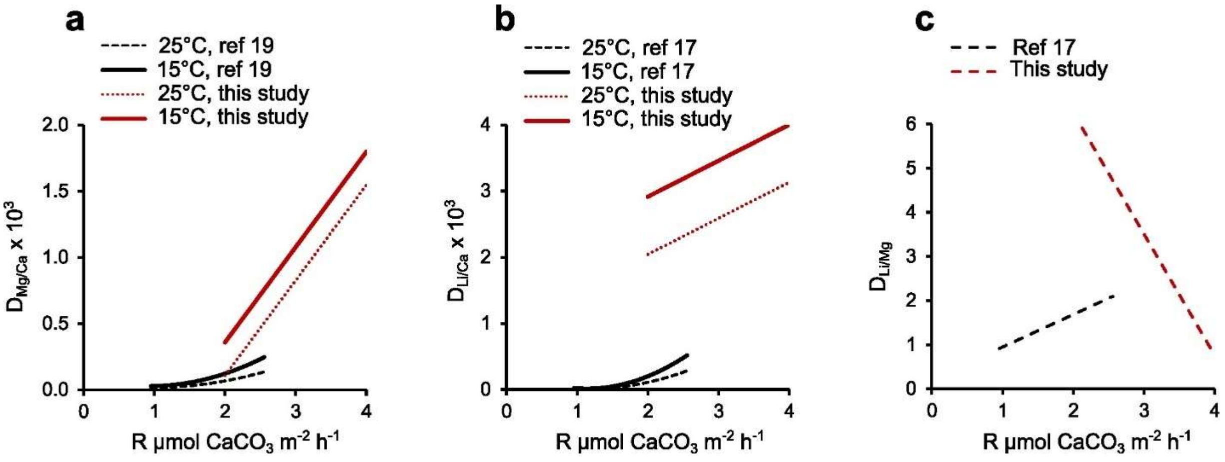

Supplementary Table 1. Details of precipitations, seawater and solid chemistry and distribution coefficients in experiments at 25°C with and without amino acid (additive).

Experiment			Experiment DIC conditions				Seawater metal chemistry				Solids chemistry			Distribution coefficients		
Our reference	Additive if used	Seawater batch	Mean DIC µmol/kg	pH total	[CO32-] µmol/kg	Ωaragonite	Final seawater [Ca] mmol/kg	Final seawater [Li] µmol/kg	Final seawater [Mg] mmol/kg	Precipitation rate micromol CaCO3/m2/h-1	Solid Li/Ca µmol/mol	Solid Mg/Ca mmol/mol	Solid Li/Mg mmol/mol	$D_{Li/Ca} \times 10^3$	$D_{Mg/Ca} \times 10^3$	$D_{Li/Mg}$
**Precipitation conditions, chemistry of seawater at end of experiments and chemistry of precipitates**																
**No amino acid**																
A1	none	1	2400	8.200	350	5.4	9.90	12.12	54.07	155	4.00	3.13	1.27	2.11	0.39	5.58
B2	none	1	1180	8.408	255	4.0	9.96	12.10	54.42	190	3.80	3.44	1.11	1.86	0.49	4.34
C2	none	1	1186	8.590	351	5.3	9.27	11.85	53.34	180	3.98	4.13	0.96	2.06	0.68	3.27
D2	none	1	2850	8.200	415	6.3	9.55	12.13	54.45	650	4.28	4.14	1.03	2.47	0.69	3.79
D3	none	1	2895	8.200	422	6.5	9.84	12.09	54.31	600	4.28	3.66	1.17	2.51	0.55	4.83
D1	none	2	2891	8.200	421	6.4	9.68	12.21	54.83	600	4.40	4.30	1.02	2.64	0.74	3.70
E2	none	2	2017	8.408	436	6.6	9.60	11.81	53.70	725	4.22	4.49	0.94	2.42	0.80	3.10
E1	none	1	2060	8.408	446	6.9	9.90	12.07	53.97	700	4.46	3.77	1.18	2.76	0.59	4.91
G1	none	1	4325	8.200	631	9.7	9.83	12.47	55.03	1820	4.41	4.87	0.91	2.65	0.91	2.80
G4	none	1	4325	8.200	631	9.7	9.78	12.17	54.44	1400	4.34	4.72	0.92	2.56	0.87	2.92
H2	none	1	2990	8.408	647	10.3	10.43	12.42	55.38	1740	4.26	4.32	0.98	2.51	0.77	3.40
I1	none	1	2197	8.590	650	10.0	9.74	12.13	54.55	1720	4.38	5.25	0.83	2.62	1.03	2.28
I3	none	1	2080	8.590	615	9.4	9.66	12.23	54.62	1600	4.35	4.80	0.91	2.57	0.89	2.83
J1	none	1	5600	8.200	816	12.4	9.61	11.92	53.40	2400	4.53	5.44	0.83	2.84	1.09	2.27
J3	none	1	5600	8.200	816	12.5	9.64	12.13	54.65	2500	4.29	5.60	0.77	2.49	1.13	1.77
K1	none	1	3559	8.408	770	11.7	9.59	12.27	54.65	2650	4.59	6.18	0.74	2.87	1.30	1.58
K3	none	1	3693	8.408	799	12.6	10.29	12.10	54.09	2400	4.24	5.05	0.84	2.50	1.00	2.31
L1	none	1	2625	8.590	776	12.1	10.03	12.28	55.13	2400	4.58	5.06	0.90	2.92	0.98	2.81
L2	none	1	2657	8.590	786	12.1	9.86	12.41	56.02	2550	4.47	5.64	0.79	2.74	1.14	1.98
M1	none	1	7800	8.200	1137	17.2	9.44	11.81	52.70	4200	4.28	6.81	0.63	2.48	1.50	0.73
M3	none	1	7800	8.200	1137	17.3	9.59	11.98	53.48	4200	4.25	6.67	0.64	2.44	1.46	0.79
N1	none	1	5058	8.408	1094	16.6	9.48	12.03	53.86	3550	4.88	6.44	0.76	3.28	1.38	1.70
N4	none	1	5222	8.408	1129	17.0	9.33	11.97	54.12	3800	4.79	7.15	0.67	3.14	1.57	1.05
O1	none	1	3759	8.590	1112	17.1	9.78	12.05	54.06	3800	4.58	6.23	0.73	2.91	1.33	1.53
O3	none	1	3796	8.590	1123	17.5	10.05	12.25	54.94	4000	4.36	6.23	0.70	2.63	1.34	1.27
**Aspartic acid**																
AAG asx1	2 mM aspartic acid	1	3177	8.200	463	6.8	9.00	13.08	53.48	262	5.54	4.05	1.27	3.90	0.65	5.32
AAG asx3	2 mM aspartic acid	1	3142	8.200	458	6.8	9.09	12.77	52.25	222	5.36	4.13	1.25	3.74	0.68	5.19
AAG asx5-2	2 mM aspartic acid	1	2137	8.408	462	7.0	9.37	15.57	52.20	315	5.59	4.38	1.19	3.68	0.77	4.25
AAG asx7	2 mM aspartic acid	1	1811	8.590	536	8.0	9.26	12.83	52.22	365	5.09	4.37	1.12	3.40	0.76	4.25
AAG asx9	2 mM aspartic acid	1	1686	8.590	499	7.4	9.12	12.59	51.55	340	5.28	4.29	1.13	3.67	0.73	4.33
AAG asx13	2 mM aspartic acid	1	3153	8.408	682	10.2	9.31	15.42	52.28	700	5.80	4.47	1.21	3.94	0.79	4.44
AAG asx15-2	2 mM aspartic acid	1	2667	8.408	577	8.5	9.00	15.32	51.56	600	5.88	5.25	1.05	3.99	1.02	3.37
AAG asx16	2 mM aspartic acid	1	2085	8.590	617	9.3	9.34	12.91	52.48	750	5.37	4.74	1.08	3.78	0.87	3.96

Supplementary Table 1. Details of precipitations, seawater and solid chemistry and distribution coefficients in experiments at 25°C with and without amino acid (additive).

Experiment			Experiment DIC conditions				Seawater metal chemistry				Solids chemistry			Distribution coefficients		
Our reference	Additive if used	Seawater batch	Mean DIC µmol/kg	pH total	[CO32-] µmol/kg	Ωaragonite	Final seawater [Ca] mmol/kg	Final seawater [Li] µmol/kg	Final seawater [Mg] mmol/kg	Precipitation rate micromol CaCO3/m2/h-1	Solid Li/Ca µmol/mol	Solid Mg/Ca mmol/mol	Solid Li/Mg mmol/mol	$D_{Li/Ca} \times 10^3$	$D_{Mg/Ca} \times 10^3$	$D_{Li/Mg}$
**Precipitation conditions, chemistry of seawater at end of experiments and chemistry of precipitates**																
**No amino acid**																
AAG asx22	2 mM aspartic acid	1	3830	8.408	828	12.2	8.98	15.01	50.98	1280	6.21	5.43	1.07	4.41	1.08	3.54
AAG asx24	2 mM aspartic acid	1	3825	8.408	827	12.2	8.92	14.89	50.42	1300	6.42	5.49	1.10	4.66	1.10	3.72
AAG asx26	2 mM aspartic acid	1	2770	8.590	819	12.2	9.18	12.59	51.75	1270	5.59	5.21	1.03	4.08	1.01	3.62
AAG asx27	2 mM aspartic acid	1	2807	8.590	830	12.4	9.23	15.26	51.84	1300	6.58	5.30	1.17	4.86	1.04	4.18
AAG asx31	2 mM aspartic acid	1	5420	8.408	1172	17.0	8.67	12.50	51.64	2200	5.40	6.42	0.79	3.75	1.34	1.90
AAG asx32	2 mM aspartic acid	1	5310	8.408	1148	16.9	8.89	12.43	51.17	2100	5.69	6.40	0.84	4.18	1.36	2.25
AAG asx34	2 mM aspartic acid	1	3785	8.590	1120	16.5	9.00	14.83	50.12	2200	6.04	6.63	0.84	4.25	1.45	2.00
AAG asx35	2 mM aspartic acid	1	3863	8.590	1143	16.8	8.94	14.75	50.56	2100	6.29	6.54	0.90	4.53	1.41	2.41
**Glutamic acid**																
AAG glx 04	2 mM glutamic acid	2	3693	8.590	1092	15.5	8.76	14.65	50.69	2900	4.91	6.47	0.73	2.53	1.38	1.16
AAG glx05	2 mM glutamic acid	2	3727	8.590	1102	15.5	8.61	14.61	50.06	2850	5.19	6.30	0.76	2.80	1.33	1.31
AAG glx9	2 mM glutamic acid	1	1541	8.590	456	6.7	8.98	11.66	47.27	784	4.77	3.60	1.29	3.09	0.54	5.44
AAG glx 17	2 mM glutamic acid	1	2091	8.590	618	9.0	8.62	14.09	48.79	1200	5.28	4.60	1.10	3.37	0.83	3.78
AAG glx 22	2 mM glutamic acid	2	3800	8.408	822	11.7	8.85	14.73	50.57	1700	5.15	5.38	0.93	2.78	1.06	2.27
AAG glx D5	2 mM glutamic acid	2	2996	8.200	437	6.3	9.00	14.93	51.87	440	5.26	3.49	1.46	2.90	0.49	5.38
AAG GLX D6	2 mM glutamic acid	2	2961	8.200	432	6.2	8.83	15.29	52.44	300	5.48	3.46	1.53	3.06	0.47	5.75
AAG glx E6	2 mM glutamic acid	2	2119	8.408	458	6.5	8.88	14.76	51.11	370	5.22	3.52	1.45	2.86	0.50	5.28
AAG glx F6	2 mM glutamic acid	2	1572	8.590	465	6.6	8.82	14.57	50.58	410	5.11	3.56	1.40	2.75	0.51	5.04
AAG glx G4	2 mM glutamic acid	2	4314	8.200	629	8.9	8.64	14.65	50.67	965	5.20	3.99	1.26	2.81	0.63	4.20
AAG glx M4	2 mM glutamic acid	2	8000	8.200	1166	16.3	8.45	14.22	49.28	3300	5.33	4.38	1.12	2.96	0.76	3.39
AAG glx N4	2 mM glutamic acid	2	5202	8.408	1125	16.4	9.26	14.62	49.95	3200	5.37	6.41	0.81	3.09	1.41	1.60
AAG glx N7	2 mM glutamic acid	2	5305	8.408	1147	16.3	8.82	14.45	49.86	3400	5.32	6.17	0.80	2.99	1.31	1.56
AAG glx12	2 mM glutamic acid	2	4211	8.200	614	8.6	8.51	14.76	49.74	865	5.36	4.31	1.19	2.94	0.73	3.77
AAG glx15	2 mM glutamic acid	2	2926	8.408	633	9.1	8.94	14.58	50.37	1005	5.25	4.49	1.13	2.92	0.80	3.43
AAG glx16	2 mM glutamic acid	2	2071	8.590	613	8.7	8.84	14.47	49.90	1060	5.22	4.58	1.10	2.88	0.83	3.26
AAG glx19	2 mM glutamic acid	2	5587	8.200	814	11.5	8.64	14.85	49.85	1650	5.71	5.33	1.01	3.32	1.04	2.74
AAG glx20	2 mM glutamic acid	2	5500	8.200	802	11.4	8.85	14.88	49.73	1750	5.33	4.69	1.08	2.96	0.86	3.09
AAG glx23	2 mM glutamic acid	2	3649	8.408	789	11.2	8.67	14.86	51.40	2100	5.39	5.04	0.99	2.99	0.94	2.64
AAG glx25	2 mM glutamic acid	2	2756	8.590	815	11.7	8.91	14.86	51.40	1950	5.13	5.62	0.84	2.76	1.13	1.80
AAG glx26	2 mM glutamic acid	2	2770	8.590	819	11.7	8.91	14.96	51.61	1950	5.30	5.13	0.95	2.93	0.98	2.41
AAG glx30	2 mM glutamic acid	2	7553	8.200	1101	15.5	8.61	14.37	49.65	3000	5.16	6.15	0.78	2.80	1.29	1.43
**Glycine**																
AAG gly1	2 mM glycine	2	3016	8.200	440	6.4	8.63	14.89	50.00	550	5.22	3.62	1.40	2.81	0.53	4.93

Supplementary Table 1. Details of precipitations, seawater and solid chemistry and distribution coefficients in experiments at 25°C with and without amino acid (additive).

Experiment			Experiment DIC conditions				Seawater metal chemistry				Solids chemistry			Distribution coefficients		
Our reference	Additive if used	Seawater batch	Mean DIC µmol/kg	pH total	[CO32-] µmol/kg	Ωaragonite	Final seawater [Ca] mmol/kg	Final seawater [Li] µmol/kg	Final seawater [Mg] mmol/kg	Precipitation rate micromol CaCO3/m2/h-1	Solid Li/Ca µmol/mol	Solid Mg/Ca mmol/mol	Solid Li/Mg mmol/mol	$D_{Li/Ca}$ x 10^3	$D_{Mg/Ca}$ x 10^3	$D_{Li/Mg}$
**Precipitation conditions, chemistry of seawater at end of experiments and chemistry of precipitates**																
**No amino acid**																
AAG gl4-3	2 mM glycine	2	2217	8.408	480	7.1	8.96	15.30	51.76	450	5.20	4.01	1.21	2.80	0.65	3.87
AAG gly6-2	2 mM glycine	2	2098	8.408	454	6.6	8.70	14.81	50.08	570	4.89	3.88	1.17	2.49	0.61	3.66
AAG gly7	2 mM glycine	2	1611	8.590	476	7.0	8.86	12.63	51.38	650	4.91	4.22	1.09	2.73	0.71	3.45
AAG gly8	2 mM glycine	2	1650	8.590	488	7.1	8.82	12.62	51.03	680	4.32	3.89	1.08	2.07	0.61	3.38
AAG gly 24	2 mM glycine	2	3817	8.408	825	12.0	8.64	14.75	49.93	2200	6.38	5.50	1.07	4.01	1.09	3.08
AAG gly 31	2 mM glycine	2	5181	8.408	1121	15.9	8.27	32.69	48.77	3600	7.84	7.19	1.01	3.37	1.58	1.68
AAG gly12-2	2 mM glycine	2	4246	8.200	619	9.0	8.63	14.95	49.95	1350	4.80	4.45	1.04	2.37	0.78	2.89
AAG gly12-3	2 mM glycine	2	4246	8.200	619	8.9	8.53	14.90	49.97	1350	4.69	4.11	1.10	2.25	0.67	3.25
AAG gly14	2 mM glycine	2	2731	8.408	591	8.5	8.56	15.27	51.05	1100	4.90	4.60	1.03	2.45	0.81	2.81
AAG gly15	2 mM glycine	2	2914	8.408	630	9.3	8.92	12.97	47.72	1400	4.56	4.17	1.06	2.32	0.72	3.16
AAG gly17	2 mM glycine	2	2032	8.590	601	8.7	8.70	12.61	51.25	1200	4.54	4.50	0.97	2.30	0.79	2.71
AAG gly18 this is (	2 mM glycine	2	5205	8.408	1127	16.4	8.75	12.44	46.19	3200	4.49	4.42	0.99	2.27	0.80	2.71
AAG gly19	2 mM glycine	2	5444	8.200	794	11.5	8.65	14.93	50.13	2100	5.28	4.97	0.98	2.87	0.93	2.54
AAG gly23	2 mM glycine	2	3649	8.408	789	11.3	8.40	15.02	50.19	2000	5.10	5.86	0.81	2.64	1.18	1.56
AAG gly25-2	2 mM glycine	2	2651	8.590	784	11.4	8.76	14.42	49.50	2000	5.00	5.26	0.88	2.65	1.03	2.02
AAG gly26	2 mM glycine	2	2662	8.590	787	11.2	8.26	14.63	48.33	2020	5.41	5.17	0.97	2.97	0.99	2.47
AAG gly28	2 mM glycine	2	7432	8.200	1083	15.8	8.80	15.24	51.81	3050	5.09	5.92	0.83	2.67	1.21	1.68
AAG gly29	2 mM glycine	2	7324	8.200	1068	15.4	8.52	14.94	50.22	3050	5.02	5.66	0.82	2.59	1.13	1.65
AAG gly32	2 mM glycine	2	4975	8.408	1076	15.3	8.33	32.94	49.44	3600	7.79	6.13	1.18	3.33	1.27	2.27
AAG gly34	2 mM glycine	2	3776	8.590	1117	16.1	8.51	14.89	50.31	3700	5.19	6.53	0.74	2.76	1.39	1.17
AAG gly36	2 mM glycine	2	3657	8.590	1082	15.8	8.81	15.14	51.77	3450	5.24	6.78	0.71	2.84	1.46	1.04
**Chemistry of seawater at start of experiments**																
Seawater batch 1							10.14	12.07	55.03							
Seawater batch 2							9.65	14.99	51.80							
**Chemistry of seed**																
Coral seed replicate 1											6.24	4.64	1.35			
Coral seed replicate 2											6.08	4.63	1.31			
Average coral seed											6.16	4.63	1.33			

Supplementary Table 2. Details of precipitations, seawater and solid chemistry and distribution coefficients in experiments at variable temperature. nd = not determined.

**Experiment conditions**									**Seawater metal chemistry**							**Solids chemistry**			**Distribution coefficients**		
**Our reference**	**Temperature**	**Temperature variation (1s)**	**Start DIC µmol/kg**	**Final DIC µmol/kg**	**Mean DIC µmol/kg**	**pH total scale**	**[CO32-] µmol/kg**	**Ωaragonite**	**Start seawater [Ca] mmol/kg**	**Start seawater [Li] µmol/kg**	**Start seawater [Mg] mmol/kg**	**Final seawater [Ca] mmol/kg**	**Final seawater [Li] µmol/kg**	**Final seawater [Mg] mmol/kg**	**Precipitation rate micromol CaCO3/m2/h-1**	**Solid Li/Ca µmol/mol**	**Solid Mg/Ca mmol/mol**	**Solid Li/Mg mmol/mol**	**$D_{Li/Ca}$ x 10^3**	**$D_{Mg/Ca}$ x 10^3**	**$D_{Li/Mg}$**
p2	25.05	0.04	4517	4380	4449	8.35	871	13.5	10.4	21.8	55.2	9.68	21.2	53.20	2740	5.29	4.96	1.07	2.77	1.09	2.45
p3	25.09	0.01	4477	4428	4453	8.35	873	14.0	10.7	22.1	56.2	10.10	21.6	54.21	2571	5.56	4.80	1.16	3.04	1.06	2.84
p4	25.37	0.01	4539	4281	4410	8.35	872	13.5	10.6	22.0	55.8	9.52	21.6	54.15	3216	5.65	5.66	1.00	3.00	1.29	2.16
p5	29.95	0.01	4074	3843	3959	8.33	848	13.8	10.5	21.9	55.8	9.97	21.5	54.21	4440	5.15	4.86	1.06	2.69	1.06	2.43
p6	30.27	0.03	4082	3964	4023	8.33	870	14.1	10.8	22.0	56.1	9.63	21.4	53.96	4138	5.45	5.54	0.98	2.92	1.27	2.11
p7	29.87	0.05	4016	3854	3935	8.33	841	13.8	10.6	22.0	56.1	10.11	21.5	54.02	4178	5.06	4.71	1.08	2.64	1.03	2.49
p9	10.32	0.06	6119	5803	5961	8.43	854	12.7	10.5	21.8	55.7	9.67	21.1	53.21	350	6.53	4.07	1.60	3.76	0.81	4.74
p10	10.49	0.02	6994	6745	6870	8.50	1120	16.2	10.1	20.9	53.3	9.54	21.2	53.66	722	6.64	5.25	1.27	3.81	1.17	3.31
p11	10.25	0.09	4932	nd	4932	8.36	605	9.0	10.5	22.0	56.1	9.67	21.1	52.97	147	6.60	3.27	2.02	3.80	0.57	6.48
p12	10.91	0.03	4127	4180	4154	8.28	445	7.0	10.6	21.9	55.8	10.73	21.2	53.22	57	5.40	2.82	1.92	3.03	0.45	6.04
p13	10.25	0.05	6014	5709	5862	8.43	838	12.6	10.4	21.6	55.1	9.93	21.1	53.28	351	6.48	4.08	1.59	3.76	0.83	4.67
p14	10.48	0.04	6758	6802	6780	8.50	1105	16.2	10.4	21.5	55.0	9.58	21.0	53.07	617	6.36	5.07	1.26	3.62	1.12	3.27
p15	10.23	0.02	4730	4394	4562	8.36	560	9.5	11.3	22.1	56.0	11.80	21.3	53.46	222	5.76	2.89	2.00	3.57	0.51	6.37
p17	10.22	0.03	7338	7036	7187	8.50	1161	17.3	10.5	21.8	55.6	9.74	21.0	53.49	560	6.60	4.96	1.33	3.83	1.09	3.60
p18	15.50	0.02	5354	5234	5294	8.46	941	14.3	10.6	21.9	56.0	9.75	21.4	53.77	971	6.45	5.04	1.28	3.68	1.11	3.35
p19	15.22	0.03	5465	5242	5354	8.42	874	13.3	10.6	21.8	55.9	9.83	21.2	53.80	689	6.00	4.04	1.49	3.38	0.81	4.27
p21	15.16	0.04	5200	5081	5141	8.42	837	12.8	10.6	22.4	56.6	9.87	21.1	53.82	571	6.19	3.94	1.57	3.49	0.77	4.60
p22	20.27	0.03	4235	4112	4174	8.30	633	10.3	11.0	23.0	58.6	10.60	22.5	56.47	846	5.60	3.63	1.54	3.06	0.69	4.46
p23	20.27	0.03	4252	4130	4191	8.30	635	10.1	10.9	23.1	57.8	10.05	22.6	55.85	687	5.40	3.64	1.49	2.80	0.68	4.15
p24	20.24	0.04	5702	5339	5521	8.42	1068	17.7	11.2	24.1	60.2	10.75	23.4	57.63	2258	5.89	5.44	1.08	3.20	1.24	2.47
p25	20.13	0.06	5762	5409	5586	8.42	1077	17.3	10.9	23.2	57.9	10.35	22.9	56.59	1755	6.13	5.76	1.06	3.38	1.34	2.40
p26	20.23	0.03	3410	3415	3413	8.22	443	6.9	10.5	22.4	55.8	10.00	22.2	54.51	233	5.18	2.79	1.86	2.64	0.42	5.65
p27	20.24	0.02	3411	3371	3391	8.22	441	6.9	10.7	22.7	56.0	10.16	22.2	54.71	251	5.42	2.66	2.04	2.85	0.38	6.38
p28	20.17	0.01	4985	4772	4879	8.38	867	14.1	11.2	24.2	59.9	10.26	23.3	57.21	1445	5.70	4.80	1.19	2.99	1.03	2.89
p29	20.27	0.05	5031	4874	4953	8.38	883	14.4	11.2	24.0	59.5	10.43	23.0	56.92	1540	5.72	5.13	1.11	3.05	1.14	2.60
p30	20.22	0.06	5123	4905	5014	8.38	893	15.0	11.1	23.7	59.0	nd	nd	nd	1564	5.60	4.79	1.17	3.00	1.05	2.84
p31	20.23	0.02	4234	4186	4210	8.30	637	11.0	11.4	24.5	60.9	nd	nd	nd	830	5.70	3.85	1.48	3.08	0.76	4.13
p32	20.12	0.09	5680	5377	5529	8.42	1066	17.0	10.5	22.3	55.8	nd	nd	nd	2179	5.43	5.42	1.00	2.91	1.25	2.16
p33	29.84	0.06	3449	3444	3447	8.24	630	10.1	10.3	21.8	54.3	9.90	22.3	54.96	2522	4.94	4.39	1.13	2.44	0.91	2.65
p34	29.79	0.10	3410	3282	3346	8.21	580	9.4	10.7	22.7	56.4	9.90	22.3	54.96	2191	4.83	3.83	1.26	2.35	0.74	3.19
p36	29.97	0.04	3390	3265	3328	8.24	611	10.6	10.8	22.8	57.0	11.13	25.6	63.29	2230	4.96	4.07	1.22	2.43	0.80	3.05
p37	29.78	0.08	4891	4575	4733	8.38	1116	18.8	11.8	25.2	62.9	9.53	21.7	53.72	6189	5.26	6.07	0.87	2.66	1.41	1.59
p38	29.85	0.11	2802	2830	2816	8.16	444	7.0	10.5	22.4	55.8	9.46	21.1	52.08	886	4.66	3.22	1.44	2.24	0.55	3.97
p40	29.72	0.06	2834	2791	2813	8.16	442	7.1	10.4	22.5	55.9	9.78	21.9	54.01	975	4.51	3.16	1.43	2.10	0.53	3.89
p44	10.39	0.03	5952	5812	5882	8.43	845	12.6	10.5	22.2	55.5	9.71	21.7	53.56	339	6.43	4.15	1.55	3.60	0.84	4.41
p45	10.35	0.05	4120	4107	4114	8.28	432	6.5	10.5	22.2	55.6	10.00	21.8	53.94	33	5.80	2.67	2.17	3.15	0.39	6.98
p46	10.31	0.05	4788	4759	4774	8.36	587	8.8	10.6	22.3	56.1	9.71	21.8	53.77	129	6.03	3.20	1.88	3.28	0.54	5.80
p47	29.90	0.04	5049	4775	4912	8.38	1162	18.7	10.7	22.5	56.6	9.64	21.8	54.31	5576	5.17	6.07	0.85	2.63	1.42	1.53
Synthetic seed 1																**4.33**	**3.52**	**1.23**			
Synthetic seed 2																4.48	3.55	1.26			
Synthetic seed 3																4.23	3.70	1.14			
Mean seed																4.35	3.59	1.21			

Supplementary Table 3. Expected and measured seawater metal concentrations in replicate IAPSO analyses in each analytical run.

			Ca mmol/l	Mg mmol/l	Li µmol/l	Mg/Ca mol/mol	Li/Ca mmol/mol	Li/Mg mmol/mol
Expected value			10.55	53.98	26.9			
**Run 1**		1	10.49	54.71	26.92	5.216	2.566	0.492
		2	10.47	54.63	26.88	5.217	2.567	0.492
		3	10.48	54.63	26.80	5.212	2.557	0.491
		4	10.48	54.72	26.91	5.219	2.567	0.492
		5	10.50	54.81	27.07	5.219	2.578	0.494
		6	10.50	54.73	27.08	5.214	2.580	0.495
	**Mean**		**10.49**	**54.71**	**26.94**	**5.216**	**2.569**	**0.492**
	**1 s**		**0.010**	**0.067**	**0.109**	**0.003**	**0.008**	**0.002**
**Run 2**		1	10.19	53.34	26.82	5.236	2.632	0.503
		2	10.33	54.06	27.18	5.232	2.630	0.503
		3	10.33	54.19	26.96	5.246	2.609	0.497
		4	10.22	53.58	26.53	5.245	2.597	0.495
		5	10.22	53.48	26.86	5.232	2.628	0.502
		6	10.28	53.79	27.09	5.235	2.636	0.504
		7	10.21	53.46	26.86	5.238	2.632	0.502
		8	10.33	54.12	27.10	5.239	2.623	0.501
		9	10.32	54.04	26.94	5.237	2.610	0.498
		10	10.13	53.54	27.10	5.285	2.675	0.506
	**Mean**		**10.25**	**53.76**	**26.94**	**5.243**	**2.627**	**0.501**
	**1 s**		**0.072**	**0.319**	**0.190**	**0.016**	**0.021**	**0.003**
Run 3		1	10.50	54.47	26.90	5.187	2.561	0.494
		2	10.47	54.43	26.61	5.198	2.541	0.489
		3	10.48	54.47	26.53	5.196	2.531	0.487
		4	10.50	54.45	26.83	5.184	2.555	0.493
		5	10.49	54.40	26.91	5.184	2.564	0.495
		6	10.47	54.45	27.29	5.198	2.606	0.501
		7	10.50	54.51	27.30	5.190	2.600	0.501
		8	10.50	54.43	27.13	5.185	2.584	0.498
		9	10.47	54.28	26.97	5.185	2.576	0.497
	**Mean**		**10.49**	**54.43**	**26.94**	**5.190**	**2.569**	**0.495**
	**1 s**		**0.014**	**0.064**	**0.271**	**0.006**	**0.025**	**0.005**

Supplementary Table 4. Values of $CaCO_3$ reference materials analysed with aragonite samples in each run.

	Reference material	Mg/Ca mmol/mol	Li/Ca µmol/mol	Li/Mg mmol/mol	Reference material	Mg/Ca mmol/mol	Li/Ca µmol/mol	Li/Mg mmol/mol
**Run 1**	JCp-1	4.26	6.28	1.47	NIST RM 8301	4.19	5.63	1.34
	JCp-1	4.16	6.16	1.48	NIST RM 8301	4.19	5.74	1.37
	JCp-1	4.22	6.33	1.50	NIST RM 8301	4.18	5.64	1.35
	JCp-1	4.19	6.36	1.52	NIST RM 8301	4.15	5.57	1.34
	JCp-1	4.19	6.53	1.56	NIST RM 8301	4.14	5.63	1.36
	JCp-1	4.19	6.17	1.47	NIST RM 8301	4.14	5.56	1.34
	JCp-1	4.16	6.23	1.50	NIST RM 8301	4.11	5.58	1.36
	JCp-1	4.23	6.28	1.48	NIST RM 8301	4.10	5.57	1.36
	JCp-1	4.26	6.35	1.49	NIST RM 8301	4.12	5.56	1.35
	JCp-1	4.26	6.15	1.44	NIST RM 8301	4.13	5.46	1.32
	JCp-1	4.23	6.02	1.42	NIST RM 8301	4.07	5.48	1.35
	JCp-1	4.22	6.12	1.45	NIST RM 8301	4.08	5.42	1.33
	JCp-1	4.26	6.09	1.43	NIST RM 8301	4.08	5.41	1.33
	**Average**	**4.218**	**6.236**	**1.478**	**Average**	**4.128**	**5.558**	**1.346**
	**1 s**	**0.036**	**0.137**	**0.037**	**1s**	**0.042**	**0.095**	**0.014**
**Run 2**	JCp-1	4.15	6.29	1.52	NIST RM 8301	3.99	5.55	1.39
	JCp-1	4.19	6.34	1.51	NIST RM 8301	3.99	5.46	1.37
	JCp-1	4.19	6.28	1.50	NIST RM 8301	3.97	5.62	1.41
	JCp-1	4.16	6.16	1.48	NIST RM 8301	3.95	5.52	1.40
	JCp-1	4.07	6.22	1.53	NIST RM 8301	3.94	5.46	1.38
	JCp-1	3.97	6.08	1.53	NIST RM 8301	3.87	5.24	1.35
	JCp-1	3.91	6.12	1.56	NIST RM 8301	3.86	5.38	1.39
	JCp-1	3.92	6.08	1.55	NIST RM 8301	3.88	5.17	1.33
	JCp-1	3.89	6.02	1.55	NIST RM 8301	3.86	5.55	1.44
	JCp-1	3.86	5.93	1.53	NIST RM 8301	3.84	5.24	1.36
	**Average**	**4.033**	**6.152**	**1.526**	**Average**	**3.916**	**5.418**	**1.384**
	**1 s**	**0.135**	**0.130**	**0.026**	**1 s**	**0.058**	**0.156**	**0.032**
**Run 3**					NIST RM 8301	4.00	5.10	1.28
					NIST RM 8301	4.06	5.16	1.27
					NIST RM 8301	4.01	5.09	1.27
					NIST RM 8301	4.06	5.38	1.32
					NIST RM 8301	4.05	5.33	1.32
					NIST RM 8301	4.04	5.23	1.29
					NIST RM 8301	4.09	5.24	1.28
					NIST RM 8301	4.02	5.24	1.30
					NIST RM 8301	4.05	5.33	1.32
					**Average**	**4.042**	**5.232**	**1.294**
					**1 s**	**0.029**	**0.104**	**0.021**

Supplementary Table 5. Equations describing $D_{Me/Me}$ as a function of aragonite growth rate (R, µmol m^{-2} h^{-1}) and pH (total scale) in precipitations conducted at 25°C in the presence of 2 mM aspartic acid, glutamic acid or glycine. Coefficients of determination (r^2) and p values for each equation coefficient are shown. p values ≥0.05 are highlighted in bold. Standard errors of equation coefficients and intercepts are included in brackets.

Equation	r^2	p value		
**DMe/Me as a function of log precipitation rate (R) and pH at 25°C with aspartic acid (n=16)**		*rate*	*pH*	*intercept*
$D_{Mg/Ca}$ = 7.39 (±0.80) x 10^{-4} log *R* – 1.33 (±2.10) x 10^{-4} *pH* – 2.09 (±170) x 10^{-5}	0.88	**4.5x10^{-7}**	0.54	0.99
$D_{Li/Ca}$ = 8.03 (±2.69) x 10^{-4} log *R* – 3.85 (±7.09) x 10^{-4} *pH* + 5.05 (±5.71) x 10^{-3}	0.42	**0.011**	0.60	0.39
$D_{Li/Mg}$ = -2.62 (±0.46) *R* - 0.197 (± 1.20) *pH* + 13.0 (±9.7)	0.76	**6.8x10^{-5}**	0.87	0.20
**DMe/Me as a function of log precipitation rate (R) and pH at 25°C with glutamic acid (n=22)**				
$D_{Mg/Ca}$ = 8.12 (±0.99) x 10^{-4} log *R* + 2.46 (±1.92) x 10^{-4} *pH* - 3.71 (±1.61) x 10^{-3}	0.79	**1.2x10^{-7}**	0.22	**0.033**
$D_{Li/Ca}$ = -4.52 (±13.5) x 10^{-5} log *R* – 1.49 (±2.63) x 10^{-4} *pH* + 4.33 (±2.20) x 10^{-3}	0.02	0.74	0.58	0.064
$D_{Li/Mg}$ = -4.05 (±0.41) log *R* - 0.949 (± 0.802) *pH* + 23.8 (±6.7)	0.84	**7.2x10^{-9}**	0.25	**2.2x10^{-3}**
**DMe/Me as a function of log precipitation rate (R) and pH at 25°C with glycine (n=22)**				
$D_{Mg/Ca}$ = 8.70 (±1.14) x 10^{-4} log *R* + 3.34 (±2.23) x 10^{-4} *pH* – 4.65 (±1.91) x 10^{-3}	0.76	**3.5x10^{-7}**	0.15	**0.025**
$D_{Li/Ca}$ = 5.10 (±3.16) x 10^{-4} log *R* + 6.30 (±61.7) x 10^{-5} *pH* + 5.41 (±53.0) x 10^{-4}	0.12	0.12	0.92	0.92
$D_{Li/Mg}$ = -2.74 (±0.35) log *R* – 1.36 (± 0.68) *pH* + 22.9 (±5.8)	0.78	**2.0x10^{-7}**	0.058	**8.8x10^{-4}**

Supplementary references

1. Mavromatis, V, Brazier JM, Goetschl KE., 2022. Controls of temperature and mineral growth rate on Mg incorporation in aragonite. Geochim. Cosmochim. Acta, 317, 53-64.
2. Brazier JM, Harrison AL, Rollion-Bard C, Mavromatis V. Controls of temperature and mineral growth rate on lithium and sodium incorporation in abiotic aragonite. Chemical Geology. 2024 Jun 5;654:122057.
